# Estimation of Crack Tip Position in Adhesively Bonded Joints Subjected to Mode II Fatigue Loading

**DOI:** 10.3390/s24237676

**Published:** 2024-11-30

**Authors:** M. Mehrabi, L. M. Martulli, A. Bernasconi, M. Carboni

**Affiliations:** Department of Mechanical Engineering, Politecnico di Milano, 20156 Milano, Italy; mohammad.mehrabi@polimi.it (M.M.); lucamichele.martulli@polimi.it (L.M.M.); andrea.bernasconi@polimi.it (A.B.)

**Keywords:** adhesive bonded joints, fatigue crack propagation, mode II loading, crack length estimation, optical backscatter reflectometry, digital image correlation

## Abstract

Interest in adhesively bonded joints has significantly increased due to their numerous advantages over other joining techniques. However, they are frequently used in structures subjected to fatigue loading, which might cause defects such as cracks within the bondline. Thus, timely detection, localization, and size estimation of such defects are crucial for ensuring structural safety. This study focused on experimentally investigating crack length estimation in adhesively bonded joints under mode II fatigue loading. To analyze the crack growth, a comprehensive comparison was conducted between various techniques, such as visual testing, digital image correlation, optical backscatter reflectometry, and the analytical compliance-based beam method. In interrupted fatigue tests (static acquisition), digital image correlation and optical backscatter reflectometry exhibited consistent damage sensitivity, estimating larger crack lengths compared to visual testing by approximately 3 mm and 5 mm, respectively. The optical backscatter reflectometry in uninterrupted tests (dynamic acquisition) showed significantly larger estimations, approximately double those of static ones. This demonstrated its potential to detect possible damage within the adhesive that might not be detected by other methods, as shown previously for quasi-static loading conditions. Its capability in early damage detection under the dynamic regime makes it a valuable tool for continuous monitoring. Furthermore, a comparison of optical backscatter reflectometry’s performance in quasi-static, static, and dynamic acquisitions indicated a potentially larger process zone under quasi-static loading, a finding confirmed by the compliance-based beam method.

## 1. Introduction

Adhesive bonding has become a widely adopted joining method in various industries, particularly in aerospace and automotive sectors. These industries prioritize a combination of reliability, strength, fatigue resistance, and lightness of structures [1]. While adhesive joints excel in meeting these requirements, understanding their complex behavior under various loading conditions remains a challenge.

These joints typically experience a combination of different loading modes, ranging from pure mode I to pure mode II, as well as mixed modes. Mode I loading (opening mode) results in a crack opening perpendicular to the crack plane, while mode II loading (shearing mode) causes crack faces to slide relative to each other. Mode I loading, being the most common and detrimental type of load, has been the focus of extensive research [2]. However, mode II loading can also play a significant role in the failure of a joint, as demonstrated by several studies [3,4,5]. Consequently, understanding and considering mode II is essential in the design of reliable adhesive bonding systems, especially under fatigue loading conditions.

The process of fatigue testing for adhesive joints is often more complicated than quasi-static tests [6]. A major challenge of the fatigue test is accurately measuring the length of the crack, particularly under mode II loading conditions. Some techniques based on Structural Health Monitoring (SHM) and Non-Destructive Testing (NDT) have been developed to inspect and characterize the crack in adhesive joints under mode II loading. These include methods that mostly rely on visual observation [7,8,9], with a few utilizing elastic waves [10,11,12] that have yet to be fully developed. Visual testing (VT), the only standardized method [13] among these, is the most established and straightforward technique to monitor crack growth. Nevertheless, it has some limitations in mode II loading [14]. For instance, the lack of crack opening during propagation can make it difficult to precisely locate the crack tip position. Additionally, the dependence on the observer’s interpretation might affect the accuracy of the measurements. Moreover, continuous crack monitoring by VT can be cumbersome for long-run fatigue tests and requires interrupting the test. To address these challenges, analytical-based approaches such as the compliance-based beam method (CBBM) have been introduced [15]. The CBBM takes advantage of beam theory and the evolution of the specimen’s compliance during the test to estimate an equivalent crack length, thus obtaining fracture energy. However, it was shown that some discrepancy exists between the estimated equivalent crack length and the actual crack length measured experimentally [3,6].

Digital image correlation (DIC) is a well-established technique that enables the calculation of displacements and strains of a component’s surface. This technique is particularly valuable for analyzing strain distribution within bonded joints, aiding in crack detection and quantification. However, its application in Mode II loading is still in the early stages of research, with only a limited number of studies conducted in quasi-static [16] and fatigue loading [9,17] conditions. Similar to VT, DIC requires interrupting the fatigue test for crack length measurement.

Back-face strain analysis is another practical method based on strain measurement for crack monitoring in adhesive joints. The presence of a crack in a joint alters its stiffness and thereby the local strain. The position of the crack front can therefore be determined by this variation in the back-face strain [18]. To measure this strain, fiber optic sensors, particularly optical backscatter reflectometry (OBR) [19], offer a reliable solution. The OBR offers the advantage of distributed strain sensing with high spatial resolution over a length ranging from a few millimeters to a hundred meters. The successful application of OBR in adhesively bonded joints subjected to fatigue loading in mode I [20] and a mixed mode [21] has been proven, whereas its applicability in mode II loading, similar to DIC, remains an active area of research.

The purpose of this study is to experimentally investigate the crack length estimation in adhesive joints subjected to mode II fatigue loading. This study complements the previous findings [22] that focused on crack length estimation under mode II quasi-static loading, which helps fill the gap in knowledge regarding how these joints perform under real-world conditions, where both static and dynamic loadings are present. Understanding crack growth behavior under fatigue loading conditions contributes to developing more reliable and safer adhesive joint designs, leading to better predictive maintenance and early damage detection. A detailed comparison of experimental techniques such as VT, DIC, and back-face strain measurement using OBR was carried out along with CBBM to monitor fatigue crack growth in a metal-to-metal End-Notched Flexure (ENF) bonded joint during test interruptions. Additionally, the potential of OBR for continuous crack length estimation (without interrupting the test) is examined, aiding in the development of existing crack monitoring techniques for fatigue loading conditions. Finally, the performance of all considered methods is compared under both quasi-static and fatigue loading conditions, which highlights their strengths and limitations.

## 2. Experimental Procedures

### 2.1. Materials and ENF Sample Fabrication

**Figure 1:**
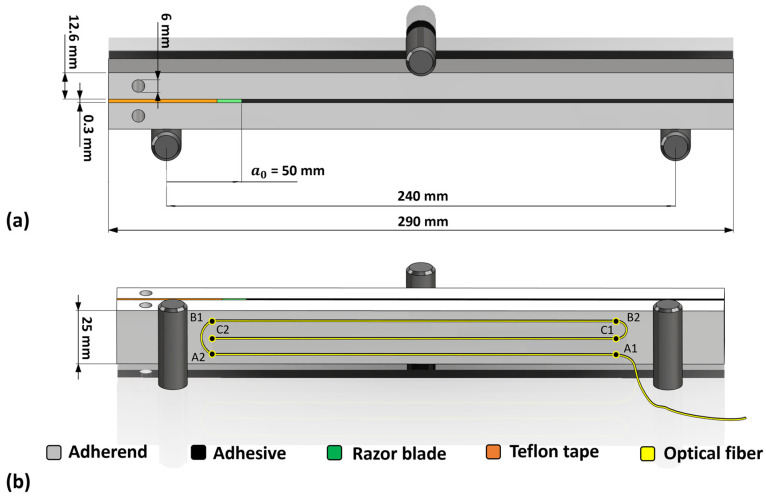
ENF sample used for three-point bending test, retrieved from [22]: (**a**) geometry of the sample in front view, (**b**) adherend back-face with three parallel paths of optical fiber bonded to its surface: path#1: A1 to A2, path#2: B1 to B2, path#3: C1 to C2. Note that drawing is not to scale.

Similar to the previous study [22], a bi-component epoxy 3M Scotch-weld^TM^ 7260B/A (3M Company, Saint Paul, MN, USA) Non-sag was used as the adhesive with a Young’s modulus of 4248 MPa, a yield stress of 35 MPa, and a Poisson’s ratio of 0.35. The substrate was a high-strength steel-grade DIN 40CrMoMn7 with a Young’s modulus of 205,000 MPa, a yield stress of 861 MPa, and a Poisson’s ratio of 0.29. The sample dimensions conformed to the ASTM D3433 standard and are illustrated in Figure 1.

This study involved two samples, S1 and S2. The manufacturing process began with sandblasting the adherends, followed by a thorough cleaning with acetone. The adhesive layer, with 0.3 mm thickness, was created by mixing adhesive with 0.3 mm diameter glass microspheres. To reduce friction during the test in the unbonded region (initial notch), a 0.15 mm thick Teflon tape was applied to the unbonded surfaces of both adherends. A razor blade was also inserted at the beginning of the bondline to sharpen the notch tip. The samples were then cured in an oven, with a temperature increase to 65 °C over 1.5 h, a steady hold at 65 °C for 3 h, and a decrease to room temperature over an hour. Any excess adhesive was removed from the sides of the ENF samples. Prior to the test, the blade was extracted from the samples.

### 2.2. Fatigue Test Configuration

The experimental fatigue tests were conducted using the three-point bending ENF test, as depicted in Figure 1a. A uni-axial MTS 810 servo-hydraulic system, equipped with a 15 kN load cell, was utilized for this purpose. The tests were performed in load control, and the fatigue load ratio (*R* = min load/max load) was set to 0.1, at a frequency of 5 Hz. The maximum load was set to 8900 N to attain 15% of the G_IIC_ value (1.2 N/mm) estimated using the J-integral approach in a previous study [22]. This value was chosen as it is high enough to cause considerable damage to the adhesive joint but low enough to allow for stable crack propagation that can be effectively tracked.

The crack propagation was monitored by periodically interrupting the test every 2000 cycles. Upon each interruption, a monotonic loading ramp with 0.5 mm/min speed was applied to achieve the maximum load, which was then held for 10 s. This allowed for the measuring of damage development using VT, DIC, and OBR. This process was repeated until the crack reached the central roller, beyond which there was insufficient driving force for further propagation. Moreover, before the fatigue test began, a static acquisition at the maximum load was established as the baseline for OBR and DIC.

### 2.3. Crack Monitoring Setup

**Figure 2:**
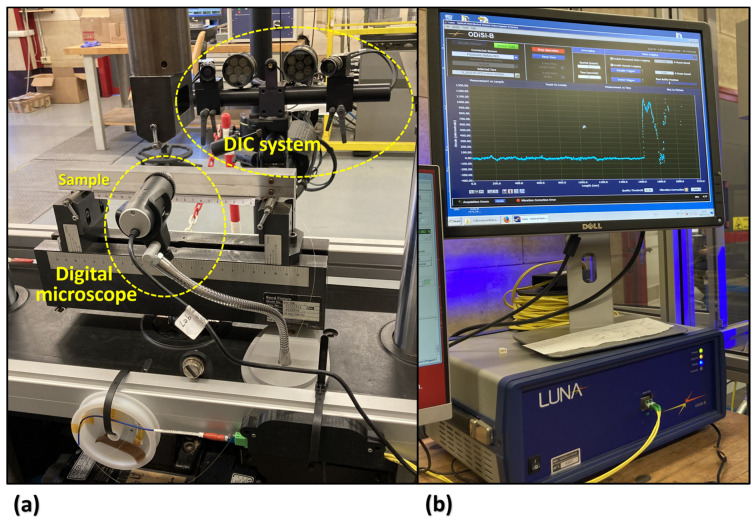
Fatigue crack monitoring setup. (**a**) The digital microscope on the back side of the specimen and DIC device on the front side. (**b**) OBR acquisition system.

The fatigue crack monitoring setup is shown in Figure 2. A high-magnification digital microscope (DinoLite, AnMo Electronics Corporation, Hsinchu, Taiwan) with an integrated LED system was used for visual monitoring of crack propagation. The VT setup was mounted on the back side of the specimen (see Figure 2a). When the test was interrupted and the ramp load reached its maximum, the frames were captured from the lateral surface at 20× magnification.

The ENF samples were prepared for DIC analysis by applying a stochastic speckle pattern followed by random sprays of black paint over a layer of white paint. For strain field measurement, the DIC system, 3D GOM ARAMIS with 12-megapixel cameras (ZEISS Compony, Oberkochen, Germany), was set up on the lateral side of the sample (see Figure 2a). The acquisition frequency was configured to 2 Hz. The region of interest was defined as 33 pixels for the subset size and 11 pixels for the step size. Post-processing was done using GOM Correlate software (ver. 2020). As the DIC device was synchronized with the testing machine, its acquisition automatically began when the fatigue loading was interrupted and continued throughout the load ramp, until the load reached its maximum value.

Similar to the configuration of OBR in the previous study [22], three parallel paths of optical fibers were implemented for back-face strain measurement. The optical fiber, with a length of 2 m, was linked to an OBR interrogator (ODiSI-B by OBR Luna Innovations Inc., Blacksburg, VA, USA). The device was then connected to a PC for the acquired data analysis, as shown in Figure 2b.

During the static acquisition (interrupted fatigue test), when the load reached its peak, strain response was acquired using a 10-sample moving average, every one second, to minimize the noise. For the dynamic acquisition (uninterrupted fatigue test), strain data was collected continuously at a frequency of 5 Hz.

## 3. Results of Interrupted Fatigue Test (Static Acquisition)

### 3.1. Visual Testing

**Figure 3:**
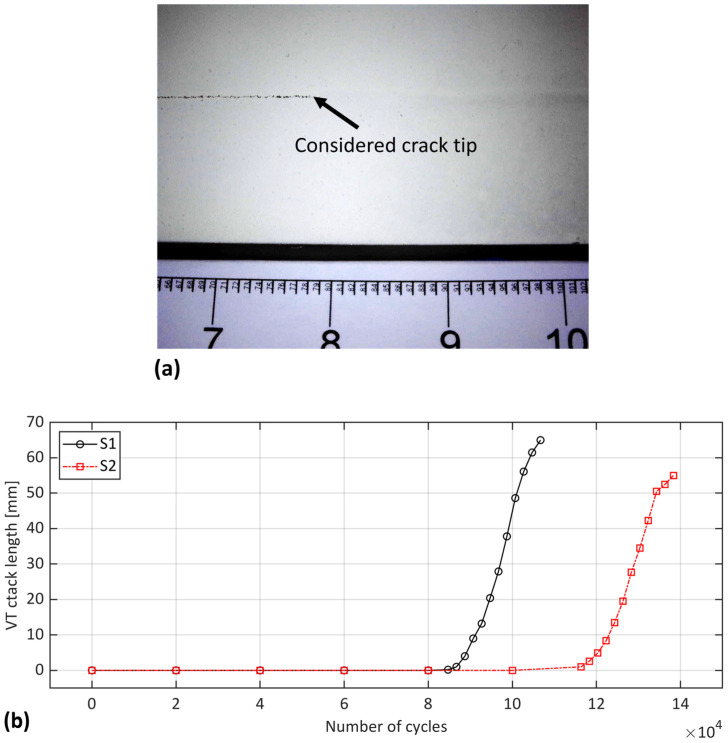
Visual testing method: (**a**) a captured frame by digital microscope during crack propagation and (**b**) measured crack length vs. number of cycles for samples S1 and S2.

The visual testing began with the capture of images using a digital microscope during fatigue test interruptions. The considered crack tip was defined as the location at which the painting layer over the bondline began to fracture, as shown by the arrow in Figure 3a. The estimated crack length included the distance from the notch tip to the identified crack tip in the analyzed frame. Figure 3b presents the estimation of crack length using the VT method at each interruption in the fatigue cycle for samples S1 and S2.

**Figure 4:**
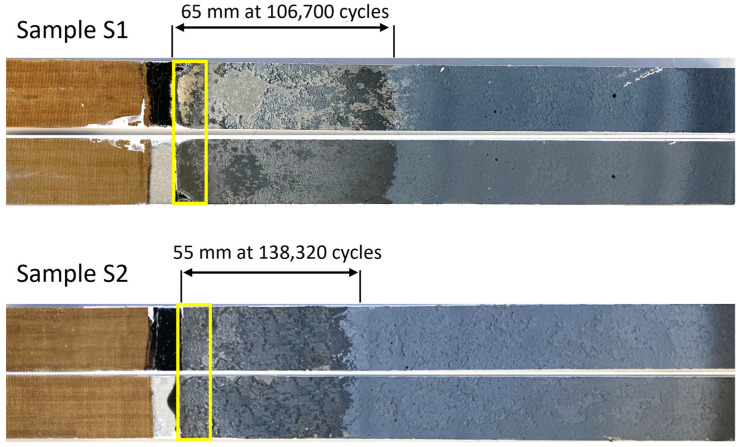
Fracture surface of specimens S1 and S2 after disassembling the samples. The yellow box highlights the distinct failure mechanisms observed in the initial stages of propagation: adhesive failure for S1 and cohesive failure for S2.

Both samples exhibited a stable crack propagation, with a similar trend in crack growth. However, there was a difference in the starting of the crack propagation between the samples: S1 began propagation slightly later than 80 k cycles, while for S2, it occurred slightly before 120 k cycles. This discrepancy could possibly be due to different failure mechanisms in the initial stages of propagation, according to Figure 4. As highlighted by the yellow box in Figure 4, sample S1 experienced adhesive failure at the beginning of the bondline, which was not observed in S2. This region of adhesive failure could explain the earlier propagation of the crack in S1. Additionally, the fracture surface of the specimens in Figure 4 shows that the primary failure mechanism within the bondline was a cohesive failure. The dark grey area with a rough surface corresponds to fatigue propagation, while the light grey area with a smooth surface represents the quasi-static disassembly of the specimen.

Beyond the observations from Figure 4, it is crucial to acknowledge other potential factors that could contribute to the varying crack initiation cycles. Material uncertainties such as voids or microcracks within the adhesive, as well as surface condition variations, and environmental effects like temperature and humidity, might have influenced the fatigue behavior of the adhesive joints [23].

Despite its limitations and subjectivity, VT was utilized as a baseline for estimating the length of cracks in the subsequent sections. This is primarily because VT is the only method that is widely accepted and standardized for the direct and straightforward measurement of crack length in adhesively bonded joints [13].

### 3.2. Digital Image Correlation

In the three-point bending ENF test, the adhesive layer, having a lower elastic modulus than the steel adherends, undergoes significant shear deformation, resulting in higher strain values. Thus, analyzing the shear strain along the adhesive layer could provide valuable insight into the location of the crack tip. This information can be extracted either directly or indirectly.

The direct method involves placing an acquisition line over the adhesive bondline and analyzing the shear strain distribution along this line. The transition point between the fluctuating and steady regions in the strain profile can be inferred as the potential crack tip. Identifying the precise location of the transition point is automatically done by the Akaike Information Criterion (AIC) algorithm; thus, the method is called DIC-AIC. On the other hand, in the indirect method, also known as the Separated Adherend Zone (SAZ), the relative displacement at the notch tip is measured and compared with the displacement over the bondline. In this study, the indirect method is denoted as DIC-SAZ. For more details on these techniques, readers may refer to [22].

**Figure 5:**
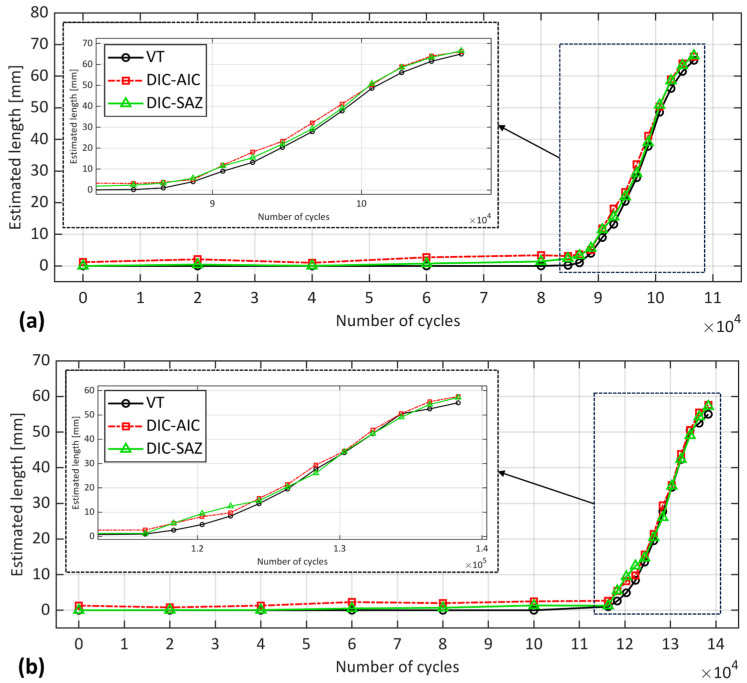
Estimated crack length of VT and DIC-based techniques for specimens (**a**) S1 and (**b**) S2. The zoomed-in part shows the crack propagation region.

By employing both the direct (DIC-AIC) and indirect (DIC-SAZ) methods, the crack length was estimated at various fatigue cycles, as shown in Figure 5. These acquisitions were made during the interruptions of the test. The findings reveal a discrepancy between DIC-AIC and VT, even at the initial load level, prior to propagation. Interestingly, DIC-AIC estimated a slightly larger crack length, where one would anticipate a length equivalent to the notch length (*a*_0_). The DIC-SAZ technique, on the other hand, was more in line with VT before propagation. After propagation, however, both methods provided similar estimations, indicating a larger crack length than VT, up to nearly 5 mm and 4 mm for S1 and S2, respectively. This overestimation was consistent with the previous findings in [9,22], suggesting that the indications by both DIC methods might not be positioned at the actual crack tip, but potentially at a location within the Fracture Process Zone (FPZ). The conformity between the two DIC methods becomes more evident in the final cycles as the crack approaches the loading point.

### 3.3. Optical Backscatter Reflectometry

**Figure 6:**
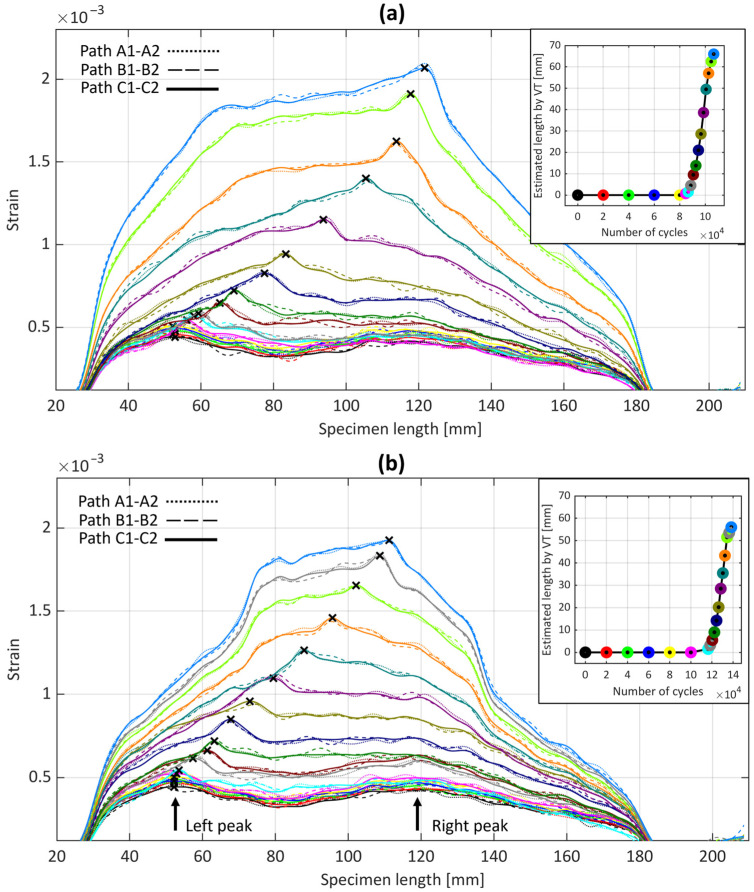
Strain distribution of three optical fiber paths bonded to the adherend at different cycles for samples (**a**) S1 and (**b**) S2.

The strain distribution variation at the back-face of ENF samples S1 and S2 were measured using the OBR technique, as represented in Figure 6. Each curve corresponds to the strain distribution at a specific cycle when the test was interrupted, differentiated by various colors.

Figure 6 reveals conformity across the three fiber paths labelled A1-A2, B1-B2, and C1-C2 (see Figure 1 for the labels). This uniformity across the paths validates the consistency of the test setup and measurements. For this reason, only the middle path (C1-C2) was considered for the subsequent analysis, due to its similarity with the other paths. Moreover, a minor fluctuating behavior in certain parts of the curves can be observed. These fluctuations could potentially be attributed to the non-uniform bonding of the fiber to the adherend, also observed in the quasi-static loading condition [22]. These fluctuating regions exhibited a similar trend as the cycle number increased, further supporting this hypothesis.

Analogous to the quasi-static loading scenario [22], the strain profile exhibited two pronounced peaks prior to the propagation of the crack. The left peaks might have resulted from the shift from a more compliant structure (unbonded arms) to a less compliant one (bonded arms). This structural shift led to a redistribution of strain, which in turn contributed to the formation of the left peaks in the strain profile. Conversely, the right peaks were due to the roller applying a concentrated load to the ENF specimen.

The joint’s stiffness was different between each interruption as the cycle count increased, leading to variations in the strain behavior. These variations were similar to those observed in the ENF adhesive joint under quasi-static loading, where the left peaks shifted rightward before crack propagation. These left peaks reflect the changes taking place during the FPZ’s development. As the cycle count rises, the crack propagates, causing the two strain peaks to converge into a single peak.

**Figure 7:**
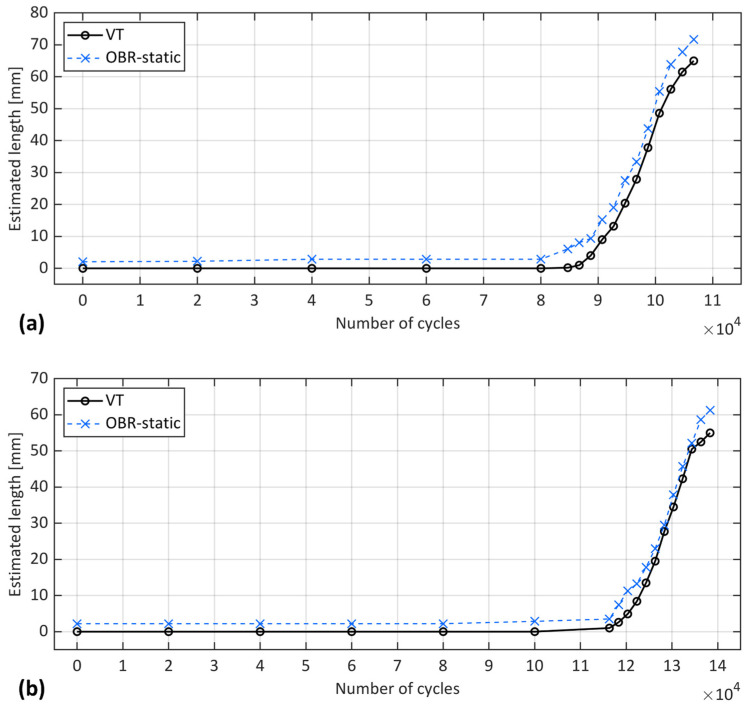
Position of strain peaks captured by OBR vs. estimated crack length of VT at different cycles for samples (**a**) S1 and (**b**) S2.

The blue crosses in Figure 7 represent the positions of strain peaks detected by OBR along the C1-C2 path, compared with the estimated lengths of VT at different cycles. The figure clearly shows that the lengths determined by VT are continuously behind the strain peaks from OBR. This is particularly noticeable in both samples during the fatigue crack propagation (around 8 mm) but less so during the initial cycles before crack propagation (around 3 mm). A plausible explanation for this observation, as also observed in the previous study [22], is that OBR is affected by the presence of a non-negligible process zone. Therefore, the OBR’s strain peaks might not correspond to the actual crack tip but rather to a point within the process zone.

### 3.4. Compliance-Based Beam Method

**Figure 8:**
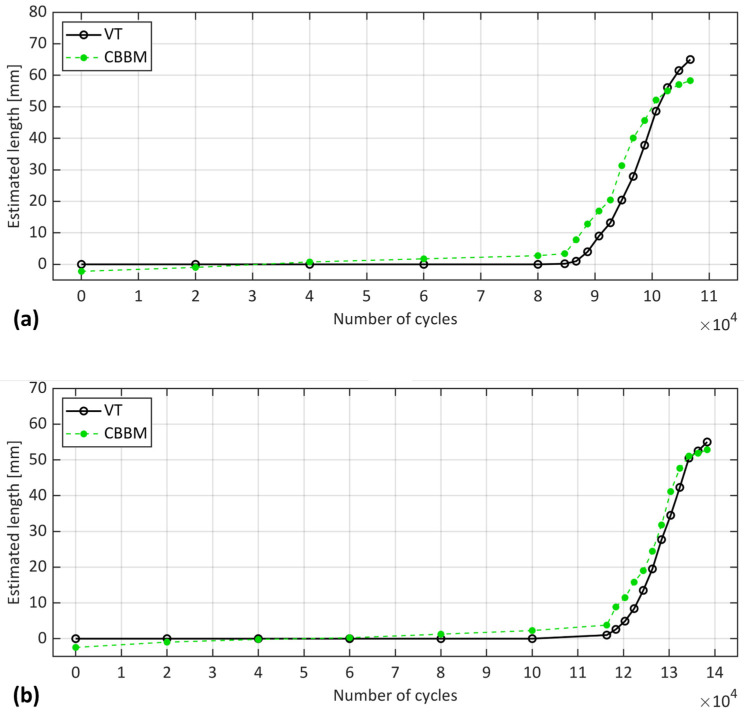
Crack length estimation using CBBM in comparison to VT at different load levels for specimens (**a**) S1 and (**b**) S2.

The equivalent crack length, an analytical concept derived from the CBBM approach, serves as a traditional alternative to experimental techniques for estimating crack length under fatigue loading conditions. This concept was therefore employed here to evaluate its efficacy in this context. In this vein, the compliance of the ENF samples was measured during the interruption of the fatigue test at the static load ramp. The results of the equivalent crack length estimated by CBBM compared with VT are shown in Figure 8.

Figure 8 shows that for most fatigue cycles, the CBBM estimated a larger crack length than the VT, except during the initial and final stages. For instance, in samples S1 and S2, the CBBM estimated a negative crack length from the first to the 30,000th cycle, where a length equivalent to the notch length was expected. This inconsistency was also observed in previous studies [22,24,25], highlighting the limitation of this method during the initial stages. Furthermore, when the crack approached the loading point in the final cycles, the CBBM estimated a smaller crack length than the VT in both samples. Such a discrepancy between CBBM and VT was also reported in [9]. Indeed, near the loading point, a highly stressed region exists under the roller’s compressive load. This region might affect the damage mechanisms within FPZ and the extent of plasticization, leading to a non-uniform distribution of stress that disrupts the expected estimation. Consequently, discrepancies arose between the crack length observed by VT and the estimated length by CBBM.

On the positive side, considering the effect of the FPZ helps in obtaining a rough estimation of the presence of microcracking and potentially some level of plasticization ahead of the actual crack tip. We therefore expected to observe a larger estimation of the crack length identified by VT. This is clearly illustrated in Figure 8, where after the 40,000th cycle, the CBBM estimated that the FPZ continued to develop until just before crack propagation at the 80,000th cycle for S1 and the 100,000th cycle for S2. This estimation can be justified by the fact that the CBBM takes into account the real changes in the compliance of the ENF sample, such as FPZ development when calculating the equivalent crack length. Indeed, even without visible crack propagation, the development of the FPZ can lead to an increase in compliance. This is because the FPZ alters the adhesive properties and stress distribution, effectively ‘softening’ the adhesive material ahead of the crack tip. This can result in an increase in strain for a given stress level, subsequently increasing compliance and ultimately leading to an increase in the estimated crack length. The larger estimation of the crack length by the CBBM is apparent during fatigue propagation in Figure 8, except for the final cycles, which reached a maximum of approximately 12 mm for S1 and 8 mm for S2.

## 4. Results of Uninterrupted Fatigue Tests (Dynamic Acquisition)

**Figure 9:**
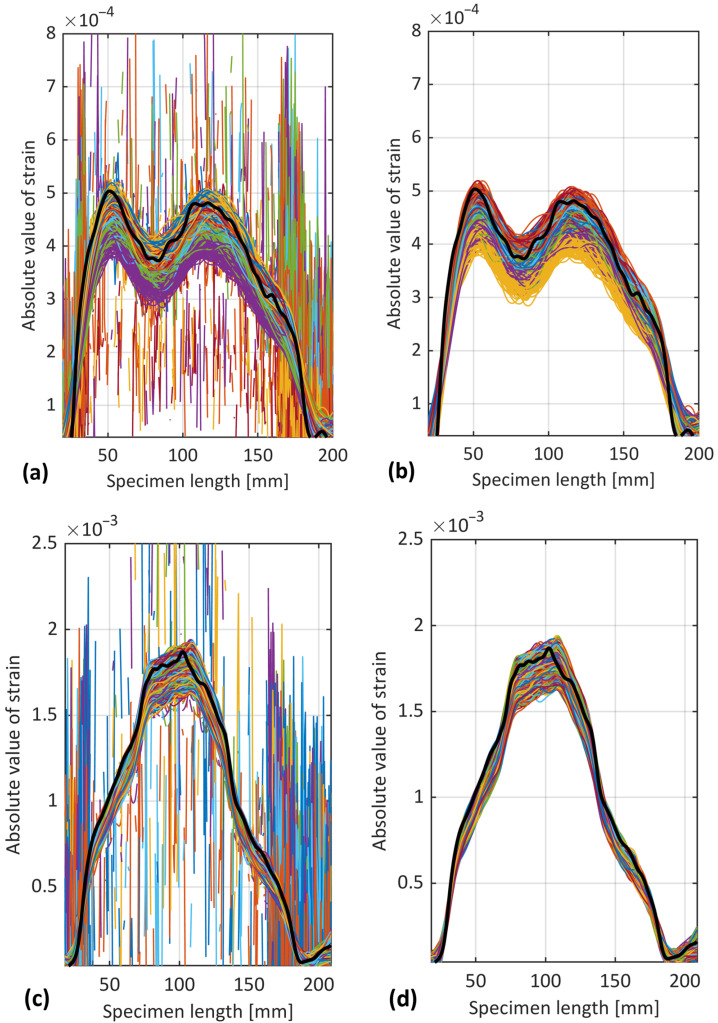
(**a**) Raw and (**b**) filtered strain signals of OBR before crack propagation (from 40,000 to 60,000 cycles). (**c**) Raw and (**d**) filtered strain signals after crack propagation (from 134,320 to 136,320 cycles) for sample S2. Black signal shows the static acquisition, and colorful signals correspond to dynamic acquisition.

The OBR was the sole method used for the fatigue tests without interruptions (dynamic acquisition). Figure 9a,c presents the raw strain signals of OBR during dynamic acquisition before and after crack propagation, respectively. Notably, these signals contained a high level of noise, which can be attributed to several factors. Firstly, to avoid losing valuable strain data under dynamic loading, additional filtering of the LUNA device was not implemented, which led to the noisy strain signals. Secondly, unlike static loading, factors such as vibrations from the testing machine under dynamic loading can affect the stability of the fiber optic sensor and the acquired data. As pointed out in [21], any noise interference within a fiber segment can lead to disregarding the acquisition for that segment, leading to incomplete and distorted signals as a consequence. Additionally, the OBR system might face challenges in accurately capturing dynamic responses, introducing subtle artifacts and noise into the acquired data. This phenomenon is common in optical fiber-based sensing systems [26]. Therefore, before evaluating the OBR performance, a filtering procedure was implemented on dynamic strain signals, as shown in Figure 9b,d. This included separating the reliable strain signal components from fragmented and distorted strain signals by identifying vectors that contained non-values in the MATLAB environment.

As observed in Figure 9, the magnitude of strain signal at static acquisition (black curve) was mainly larger than most of the dynamic acquisition (colorful curves). Interestingly, the magnitude of some dynamic signals exceeded that of the static ones, despite both being subjected to the same maximum load, both before and after crack propagation. As reported in [26], such observations are not uncommon during fatigue tests.

**Figure 10:**
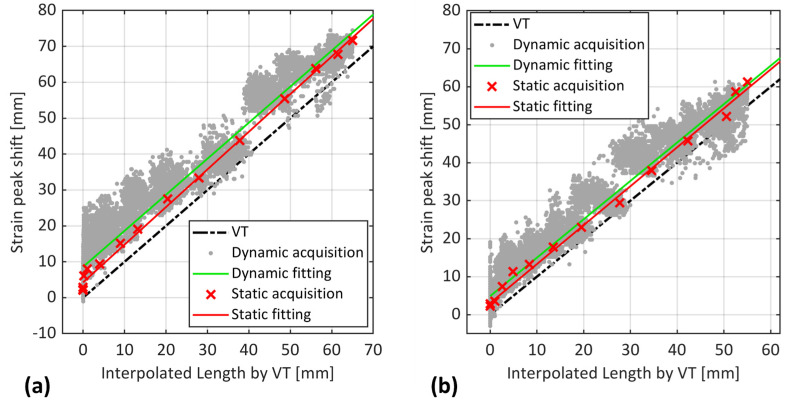
Position of strain peaks captured by OBR in both static and dynamic acquisitions vs. interpolated crack length of VT for samples (**a**) S1 and (**b**) S2.

After filtering, the dynamic and static strain peaks were compared against VT as the reference, as displayed in Figure 10. Note that the corresponding x-values for OBR dynamic acquisition were extracted from the interpolation of the VT at static acquisition.

Figure 10 reveals that, similar to static acquisition (green crosses), dynamic strain peaks (grey dots) showed an offset compared to VT, but with a larger estimation. This trend is further emphasized by the fitted line (green line), indicating an average overestimation of crack length by 10 mm for sample S1 and 3 to 6 mm for sample S2 compared to VT measurements. The discrepancy of OBR measurements between static and dynamic acquisitions is likely due to inherent differences between the loading conditions. Indeed, during static loading, the test was paused, allowing OBR to capture the strain response at the maximum load value. Under dynamic loading, however, the OBR captured the strain signals during repeated cycles of loading and unloading, where the load could be at its minimum, maximum, or somewhere in between. This constant variation in load during dynamic loading caused different strain distributions compared to static loading, as observed in Figure 9.

**Figure 11:**
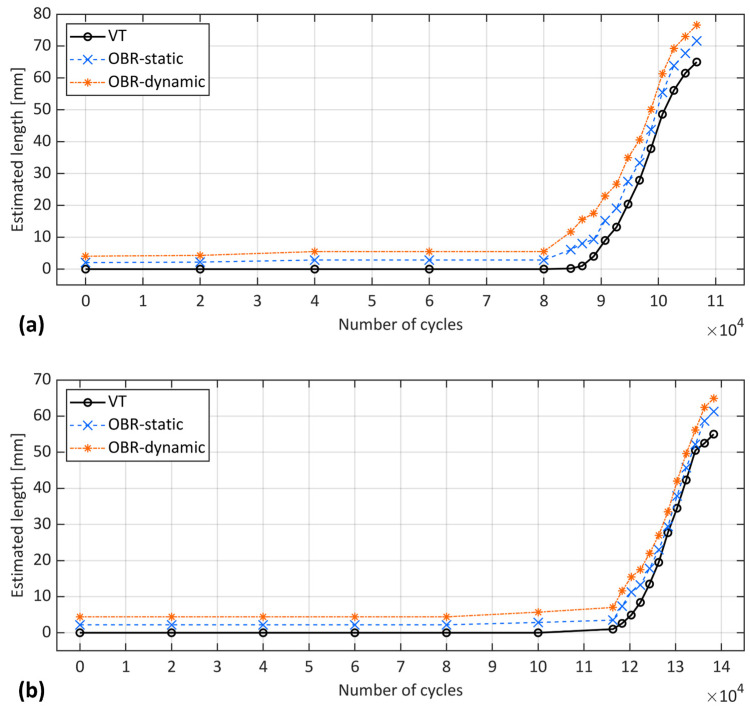
Position of strain peaks captured by OBR in static and fatigue regimes vs. estimated crack length of VT at different cycles for samples (**a**) S1 and (**b**) S2.

To enhance the comparison of OBR in static and fatigue regimes against VT throughout the cycles, datapoints from the OBR dynamic acquisition that corresponded to the number of cycles at which static acquisition were made at each interruption are identified and shown in Figure 11.

Figure 11 shows that the OBR dynamic strain peaks estimated a significantly larger crack length compared to VT and marginally larger compared to OBR at static acquisition, in agreement with the observation in Figure 10. Interestingly, this larger estimation was observed even in the initial cycles, before any crack propagation was observed in either sample according to the VT. As observed in the previous study [22], the proposed experimental methods seemed to be affected by the presence of FPZ under quasi-static loading, with OBR being more influenced than other methods. The same trend was also observed under the fatigue interrupted test (static acquisition). Therefore, one possible reason for the OBR’s overestimation could be the influence of a non-negligible process zone and the damage developed ahead of the crack tip. Another explanation could be related to the previous discussion where the repeated loading and unloading cycles in the dynamic regime caused the OBR to capture strain responses at various load levels. This could have led to a different strain distribution compared to static loading, potentially influencing the OBR’s estimation.

## 5. Discussion

### 5.1. Comparison of the Considered Methods for Crack Length Estimation

**Figure 12:**
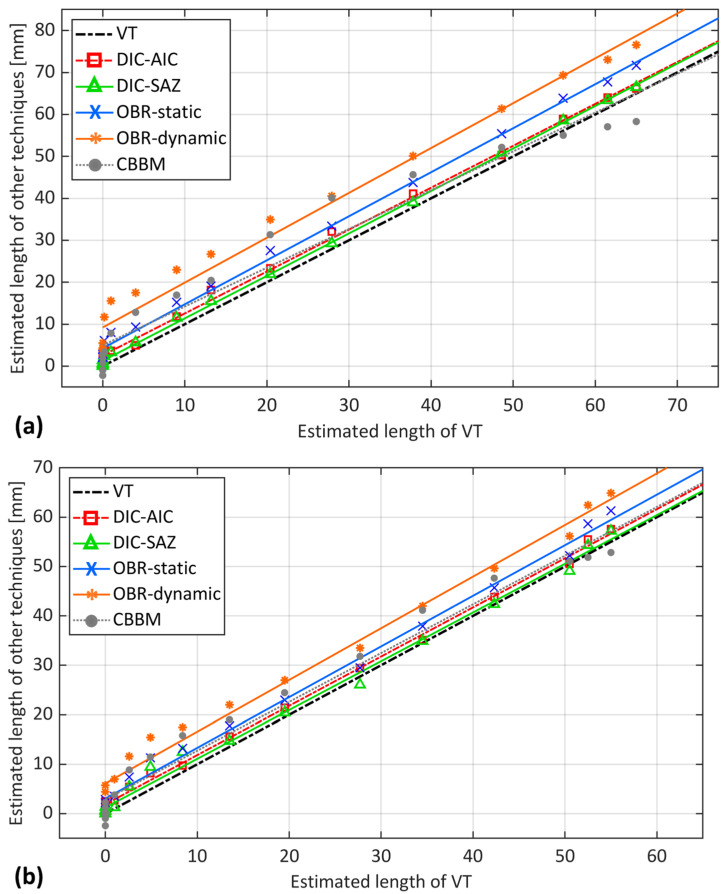
Comparison of the considered methods for fatigue crack length estimation against VT for samples (**a**) S1 and (**b**) S2.

Figure 12 shows a thorough comparison of the considered experimental and analytical methods for fatigue crack length estimation against VT for both samples. The datapoints of the methods correspond to each interruption during the test. The VT method, as a benchmark, is on the x-axis, while the other method is on the y-axis to represent a simpler comparison.

Figure 12 shows that, before crack propagation (from 0 to 5 mm on the x-axis), all the experimental techniques estimated a crack length that was larger than the identified point by the VT technique. This observation was consistent across all techniques and both samples, indicating a common trend in the early stages of crack propagation. Interestingly, the initial value for the OBR static acquisition was already about 5 mm ahead of the crack tip for both samples. The dynamic acquisition was about 8 mm to 11 mm ahead of the crack tip. In contrast, both DIC methods estimated initial values within 3 mm from the reference point at the notch tip. With the CBBM, at initial stages, a negative crack length was estimated. Under quasi-static loading, the presence of a significant process zone appeared to impact both the DIC and the OBR, with a more pronounced effect on the OBR. A similar trend was observed in the interrupted fatigue test (static acquisition), which supports the hypothesis of the possible formation of an FPZ during the initial and propagation stages and its impacts on the different techniques. The OBR in dynamic acquisition appeared to be more affected by this phenomenon. The OBR in static acquisition was slightly affected, while the DIC-AIC was less affected. The DIC-SAZ remained very close to the initial point determined by VT in both samples.

Both DIC-based approaches yielded a close estimation after crack propagation in Figure 12. DIC-AIC consistently estimated a crack length slightly greater than VT. This is observed as the fitted line for DIC-AIC remained parallel to VT, but with a 3 mm offset, for both samples. In sample S1, at the onset of propagation, DIC-SAZ estimated a crack length close to that estimated by VT. However, as the crack propagated, a divergence between these two estimates was observed. Interestingly, the opposite trend was observed for sample S2. Both DIC methods indicated a position ahead of the crack tip as identified by VT. This position was most likely associated with the process zone. Indeed, the influence of the plastic deformation and damage development in the process zone on DIC-based methods is significant enough to be detected by these methods, rather than the actual crack tip. Such an effect on the crack length estimation was also noted under quasi-static loading conditions [22].

The consequent stress redistribution due to process zone development appears to have a greater impact on the OBR than the other techniques, which is evident for both samples in Figure 12. The performance of the OBR in static acquisition for crack propagation demonstrates consistency with an estimation of about 5 to 8 mm larger than VT. The OBR in dynamic acquisition, on the other hand, showed a larger estimation than the OBR in static acquisition for both samples in Figure 12. Interestingly, there was a consistent difference between the dynamic and static acquisitions, with the dynamic estimation being approximately twice that of the static. This trend was observed in both samples. The reason for such an estimation possibly relies not only on the development of potential damage within the FPZ but also on differences in loading conditions. Indeed, the static acquisitions were performed at the maximum load level of the dynamic regime. Under dynamic loading, the OBR records strain signals throughout repeated cycles of loading and unloading, where the load could vary from its minimum to maximum or any value in between. This fluctuation in dynamic load leads to a different strain distribution compared to static loading.

Lastly, the CBBM estimation displayed the highest degree of scattering when compared to experimental methods for both samples in Figure 12. This is particularly noticeable before crack propagation, where it started with an estimation of negative crack length. Moreover, as the crack approached the loading point in the final stages, the estimation trend began to decrease, reaching even lower estimation lengths than those determined by VT. This is more evident for sample S1 in Figure 12a. Apart from the final stages, CBBM estimation during crack propagation was comparable to the estimation from the OBR in static acquisition, both identifying a point seemingly ahead of the actual crack tip. This overlap strengthens the possibility of OBR’s capability to detect a point within the potential process zone. This is because the presence of the damaged region in FPZ can lead to an increase in compliance, which in turn, results in a larger estimation from the CBBM than the actual crack length. However, discrepancies in the final stages suggest limitations in CBBM’s ability to handle the stress near the loading roller or capture the full extent of FPZ development, unlike OBR’s potential sensitivity.

From the analysis presented above, it is evident that the DIC-based techniques estimated points very close to the crack tip identified by VT. Among these, the DIC-SAZ provided an estimation closest to VT. OBR in both static and dynamic acquisitions identified a different location that was further from the estimation of both DIC and VT. When compared to VT, it could be argued that the point identified by these experimental techniques might not have been the actual crack tip itself but a point ahead of the crack tip. Indeed, the large process zone may have caused significant displacement differences in the adherends for the DIC-based methods and back-face strain redistribution for the OBR, thereby justifying a larger estimation. However, the presence of such a large process zone might not justify the OBR estimation under the fatigue regime, as it was about twice that of the static. Possible reasons for this, apart from being affected by potential damage within the FPZ, could be the differences in loading conditions. Indeed, static acquisitions were performed at the maximum dynamic load, causing a different strain distribution at each loading condition. Interestingly, the presence of a considerable process zone has been observed not only in quasi-static loading conditions but also by CBBM. In fact, CBBM considers the effect of the FPZ in its calculations, as it is based on the actual compliance of the specimen that contains a process zone within it. Consequently, this leads to results that are comparable to those of the OBR during fatigue crack propagation.

### 5.2. Comparison of Quasi-Static and Fatigue Loading Conditions

The performance of the introduced methods under both quasi-static and interrupted fatigue (static) loadings was evaluated. Additionally, the performance of OBR without interrupting the fatigue test (dynamic loading) was also examined. Data for the quasi-static tests were retrieved from a previous study [22]. The evaluation included a combination of result data from samples S1 and S2, based on which the difference in crack extension (including the crack tip and developed damage) between each loading scenario against VT was analyzed. Such an analysis enhances the understanding of the crack growth behavior and evolution of damage within the FPZ ahead of the crack tip under each loading condition.

**Figure 13:**
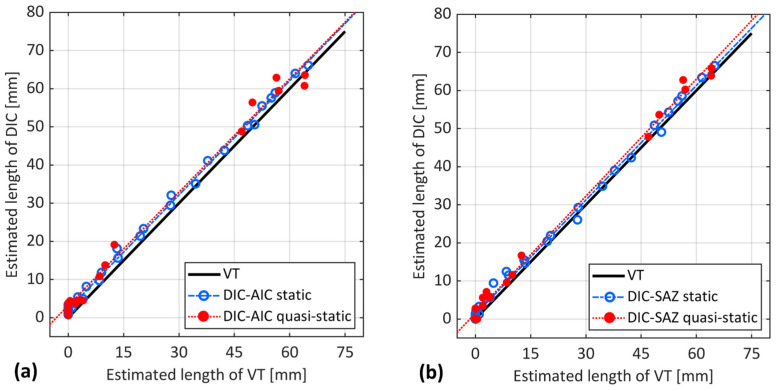

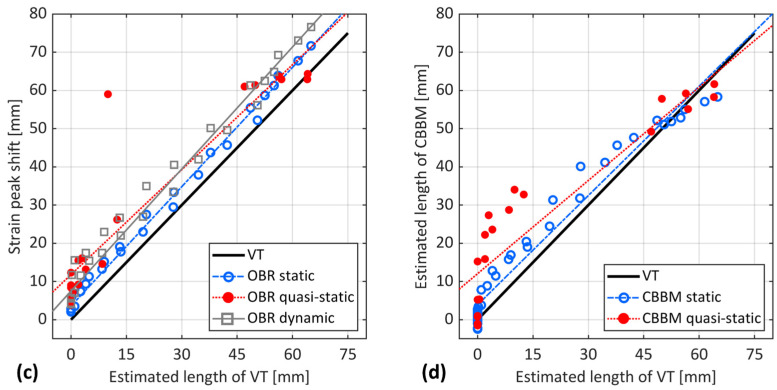
Measured crack length by VT against estimated crack length using (**a**) DIC-AIC, (**b**) DIC-SAZ, (**c**) OBR, and (**d**) CBBM under quasi-static loading, static loading (interrupted fatigue test), and dynamic loading (uninterrupted fatigue test).

Figure 13a presents the estimated crack length using the DIC-AIC method against VT under both loading conditions. It is evident that the general trend of DIC-AIC estimation under both quasi-static and static loadings was similar, consistently estimating a larger crack length of approximately 3 mm. However, DIC-AIC provided a roughly larger estimation of crack length (about 1 mm) under the quasi-static condition than in the static condition where the crack was still initiating. Moreover, the scattering of data points in the quasi-static condition, especially near the final stages, was more pronounced than in the static condition, where the results appeared smoother.

Similar to DIC-AIC, the trend of estimation of DIC-SAZ under both loading regimes exhibited a close agreement, if not identical, as depicted in Figure 13b. The figure also indicates that the DIC-SAZ method provided a marginally larger estimation of crack length in the quasi-static condition compared to the static condition. In contrast to the DIC-AIC, the datapoints of DIC-SAZ exhibit a smoother trend under fatigue loading condition, with less data point scattering. This observation might be attributed to the nature of the DIC-AIC method, which relies on the noise in strain signals. DIC-SAZ, on the other hand, seems less affected by such noise, resulting in a smoother representation of the crack length under fatigue loading.

Figure 13c clearly shows that the general trend of OBR estimation under quasi-static loading differed from static loading. The OBR in the quasi-static regime estimated a greater crack length compared to static loading. The dispersion of data points in the quasi-static condition, especially at the initial stages of propagation, is more noticeable than in the fatigue condition. At the initial stages, the difference in estimation was about 8 mm, but as the crack propagated further, the difference decreased and, interestingly, became zero at the loading point. This could be attributed to the fact that as the crack approached the loading roller, the driving force for further propagation of the crack was limited under both loading conditions. The data for dynamic loading in Figure 13c, similar to static loading, appear less scattered than the quasi-static data. Yet, it consistently provided an estimate double that of static acquisition (roughly 10 mm) compared to the VT reference values. Furthermore, the lines fitted to the data reveal a difference with the quasi-static loading at the initial stages. As the crack propagated further, there was a convergence in estimation between quasi-static and dynamic loading up to the midpoint of the bondline. Beyond this, a reverse trend was observed, with estimation under dynamic loading exceeding quasi-static loading. This divergence continued until the final stages, where the difference reached its maximum value.

The different estimations between loadings are more evident from point-by-point comparison, where the measured length using VT was about 12 mm, which was largely different from the OBR estimation in the quasi-static condition, estimating a length of 59 mm. In contrast, the same method, OBR, under static and dynamic loadings estimated a length of around 19 mm and 22 mm, respectively. The reason for such observations lies in the fact that this stage is related to a point just before unstable crack propagation in the quasi-static load, where the FPZ is developed and reaches its maximum size. As observed experimentally and numerically in previous studies [22], a significant portion of the crack propagation region within the adhesive material is occupied by the FPZ. Indeed, the difference in the load magnitude plays a role here. Under a higher load magnitude as in the quasi-static test, there could be more deformation and damage, potentially leading to a larger process zone. Conversely, under a lower load magnitude as in the static and dynamic loadings, the adhesive material might not undergo the same extent of deformation and subsequent damage, possibly resulting in a smaller process zone.

The results of CBBM in Figure 13d and OBR in Figure 13c reveal several similarities between these methods. In both methods, the estimation of quasi-static loading at initial stages, from 0 to 15 mm in VT, was significantly larger than that of fatigue loading. This corresponds to the initial stages where the FPZ region grew and reached its maximum size, which was successfully detected by both CBBM and OBR. This suggests two key points: firstly, it potentially validates the ability of OBR to detect damage development in the FPZ ahead of the crack tip, and secondly, the size of the FPZ under quasi-static loading appeared to be larger than under fatigue loading at initial stages. This observation aligns with expectations, as the size of the FPZ is considerably affected by the type and magnitude of the load [27,28]. In addition, the scattering of datapoints under quasi-static conditions was more noticeable than under static conditions for both CBBM and OBR methods. Moreover, the estimation difference between quasi-static and static loading for both methods was larger at the initial stages, around 8 mm, and gradually decreased as crack growth progressed. Interestingly, this difference converged to zero near the loading roller for both methods under both loading conditions. This observation suggests that beyond the loading point, there might be insufficient driving force to further propagate the crack under both loading conditions.

## 6. Concluding Remarks

The purpose of this paper was to experimentally investigate the crack length estimation in adhesive joints subjected to mode II fatigue loading. This study complements the previous findings that focused on crack length estimation under mode II quasi-static loading [22] and thus provides a thorough understanding of crack behavior under different loading conditions. In line with the prior study, a detailed comparison of experimental techniques such as VT, DIC, and OBR, along with the CBBM as an analytical method, was carried out to monitor fatigue crack growth. Similarly, an experimental test was conducted on the metal-to-metal ENF bonded joint using the same adherend and adhesive materials. The following results were derived based on the adhesive, loading conditions, and framework investigated in this study.

Both AIC-DIC and SAZ-DIC exhibited good repeatability and consistency in damage sensitivity. They estimated a slightly larger crack length compared to VT, with SAZ-DIC showing a closer agreement with VT. It seems that both DIC methods were influenced by the presence of the FPZ, identifying a location ahead of the crack tip measured by VT. This characteristic enhanced the value of DIC in damage identification, suggesting an early detection system for potential damage within the adhesive bondline.

OBR under static loading (interrupted fatigue test) estimated considerably larger crack lengths than the VT and DIC methods, locating a point ahead of the crack tip. This suggests that OBR was potentially more affected by the presence of a considerable process zone than DIC-based and VT, as already observed under quasi-static loading [22]. This ability of OBR makes it particularly useful for detecting early-stage damage in practical applications, potentially even at the initial stages of crack formation, before it results in failure.

Under dynamic loading (uninterrupted fatigue test), OBR consistently estimated the crack length by almost double that of static acquisition. Beyond the effect of the non-negligible process zone, the variation in load magnitude also contributed to this discrepancy. Yet, OBR was the only applicable experimental method under dynamic loading for estimating crack length with reasonable accuracy, making it a valuable tool for continuous monitoring without interrupting the test.

The comparison of OBR performance under quasi-static, static, and dynamic loadings revealed a larger crack length estimation under quasi-static loading before crack propagation, approximately 8 mm larger than static loading and 4 mm larger than dynamic loading. This confirms that the process zone is possibly larger under quasi-static loading, a conclusion that aligns with the results obtained from the CBBM.

As a common method that eliminates the complexities of experimental techniques for crack length measurement, the CBBM showed both positive and negative aspects. It considered the effect of the FPZ, which aids in obtaining a rough estimation of the crack extension within the process zone. However, its estimation exhibited high scattering compared to the experimental methods, especially at the initial and final stages of propagation. Despite this, its results aligned with OBR’s in terms of static acquisition, confirming OBR’s ability to detect damage within the process zone.

## Data Availability

Data will be available on request.
